# The Impact of Autonomic Dysfunction on Survival in Patients with Dementia with Lewy Bodies and Parkinson's Disease with Dementia

**DOI:** 10.1371/journal.pone.0045451

**Published:** 2012-10-01

**Authors:** Kajsa Stubendorff, Dag Aarsland, Lennart Minthon, Elisabet Londos

**Affiliations:** 1 Clinical Memory Research Unit, Department of Clinical Sciences, Malmö, Lund University, Sweden; 2 Center for Age-Related Medicine, Stavanger University Hospital, Stavanger, Norway; 3 Department of Neurobiology, Ward and Society, Alzheimer's Disease Research Center, Karolinska Institutet, Stockholm, Sweden; Oslo University Hospital, Norway

## Abstract

**Introduction:**

Autonomic dysfunction is a well-known feature in neurodegenerative dementias, especially common in α-synucleinopathies like dementia with Lewy bodies and Parkinson's disease with dementia. The most common symptoms are orthostatic hypotension, incontinence and constipation, but its relevance in clinical practice is poorly understood. There are no earlier studies addressing the influence of autonomic dysfunction on clinical course and survival. The aim of this study was to investigate the frequency of the three most common features of autonomic dysfunction and analyze how it affects survival.

**Methods:**

Thirty patients with dementia with Lewy bodies and Parkinson's disease with dementia were included in this prospective, longitudinal follow-up study. Presence of incontinence and constipation was recorded at baseline. Blood pressure was measured at baseline, after 3 months and after 6 months according to standardized procedures, with 5 measurements during 10 minutes after rising. Orthostatic hypotension was defined using consensus definitions and persistent orthostatic hypotension was defined as 5 or more measurements with orthostatic hypotension. Difference in survival was analyzed 36 months after baseline.

**Results:**

There was a high frequency of persistent orthostatic blood pressure (50%), constipation (30%) and incontinence (30%). Patients with persistent orthostatic hypotension had a significantly shorter survival compared to those with no or non-persistent orthostatic hypotension (Log rank x^2^ = 4.47, p = 0.034). Patients with constipation and/or urinary incontinence, in addition to persistent orthostatic hypotension, had a poorer prognosis compared to those with isolated persistent orthostatic hypotension or no orthostatic hypotension (Log rank x^2^ = 6.370, p = 0.041).

**Discussion:**

According to our findings, the identification of autonomic dysfunction seems to be of great importance in clinical practice, not only to avoid falls and other complications, but also as a possible predictor of survival.

## Introduction

Dementia with Lewy bodies (DLB) and dementia associated with Parkinson's disease (PDD) are neurodegenerative disorders with similar clinical and neuropathological features. Together they account for approximately 15–20% of all clinically diagnosed dementia cases [1], [2], [3]. Neuropathologically, they are characterized by widespread α-synuclein-containing intracytoplasmic inclusions called Lewy bodies. Lewy bodies are also the histological markers of idiopathic Parkinson's disease (PD), pure autonomic failure (PAF) and multiple system atrophy (MSA), the so called α-synucleinopaties [4].

The clinical course of DLB, PDD and all other types of neurodegenerative dementia shows a high degree of inter individual variability. There are studies reporting differences between diagnoses, where DLB seems to be a more aggressive disorder than AD [5], [6], [7] and PD [8]. Several factors to predict rapid progression and survival in DLB patients have been proposed. In a retrospective analysis of autopsy-confirmed cases with DLB, Jellinger et al found that older age at onset, fluctuating cognition, hallucinations at onset and associated AD-pathology predicted a shorter survival [9]. Boström et al found that increased levels of cerebrospinal total tau were associated with a shorter survival [10].

Autonomic dysfunction is a well-known feature in all α-synucleinopathies and in the revised diagnostic criteria for DLB it is a supportive feature. Three of the supportive features; repeated falls, syncope and transient loss of consciousness, can also be partly attributable to the presence of autonomic dysfunction. The principal autonomic symptoms are urinary incontinence, constipation and orthostatic hypotension. Autonomic dysfunction occurs to a lesser extent in AD, vascular dementia and in frontotemporal dementia [11].

Many studies have been performed on autonomic dysfunction in PD and MSA, but there is a lack of well-designed prospective studies. MSA is the α-synucleinopathy with the most pronounced autonomic dysfunction. Tada et al have shown that in MSA, an early development of autonomic dysfunction predict a poorer prognosis [12]. There is another study with the aim to test autonomic dysfunction as a predictor of survival in PD, but no correlation was found [13]. To our knowledge, this has never been studied in a DLB/PDD population.

The objective in this study is therefore to investigate the frequency of symptoms related to autonomic dysfunction (orthostatic hypotension, constipation and urinary incontinence) in a DLB/PDD population and find out whether its presence or severity is correlated to a shorter survival in these patients.

## Methods

### Subjects and study design

**Figure 1:**
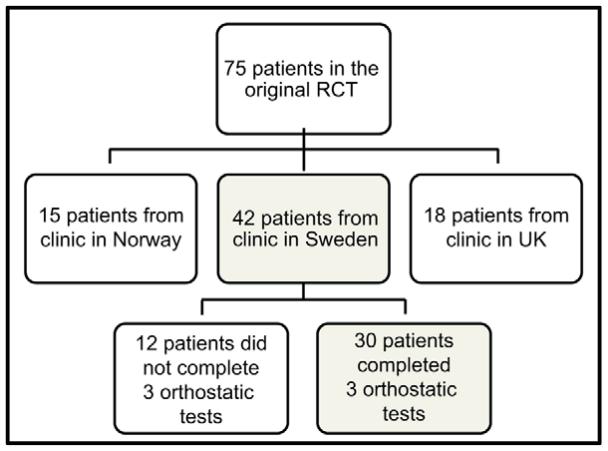
Trial profile.

This longitudinal prospective study is a continuation of a double-blind 24-week randomized placebo-controlled trial (RCT) conducted in 2005–2008 [14]. The original study included 75 patients with mild to moderate DLB or PDD (MMSE score 12 points or higher), recruited from psychiatric, memory and neurological outpatient clinics in Norway, UK and Sweden. All patients fulfilled the clinical diagnostic criteria according to UK Parkinson's disease Society Brain bank and subsequently developed dementia more than a year from onset of motor symptoms (Diagnostic and Statistical Manual of Mental disorders 4^th^ edition (DSM IV); APA, 1994) or met the revised consensus criteria for DLB [15]. The population in this study constitutes the 30 patients (16 DLB, 14 PDD) from the Swedish population (total n = 42) in the original study, who underwent assessments including orthostatic test at all three visits during the follow up (baseline, week 12 and week 24) (Figure 1).

In the original study, patients were assigned to placebo or Memantine treatment (20 mg daily) and summoned for visits at baseline, 12 and at 24 weeks. This study comprises a 4-week washout period followed by open-label treatment and ordinary yearly clinical visits within a structured follow up program at our clinic. Randomization was kept strictly double-blinded during wash out, but not during the open label treatment. Discontinuation of the double blind medication was performed by the end of the RCT without sequentially decreasing the doses. The open label medication doses were increased during a titration period of 4 weeks until reaching 20 mg daily.

In this study, outcome was recorded 36 months from baseline. Survival was the primary outcome measure.

### Blood pressure measurements

At baseline, at 12 and 24 week visits, patients completed an orthostatic test. The procedure was performed according to a standardized scheme. A validated digital spyngomanometer (OMRON M5-1) over the brachial artery was used [16]. The blood pressure and pulse rate was recorded after at least ten minutes rest in supine position, immediately after standing up and after one, three, five and ten minutes of standing. All patients stood up without assistance. Orthostatic hypotension is defined as recommended by The Consensus Committee of the American Autonomic society and the American Academy of Neurology, 1996, as a reduction in systolic blood pressure of at least 20 mmHg or a reduction of diastolic blood pressure of at least 10 mmHg.

### Orthostatic values

**Figure 2:**
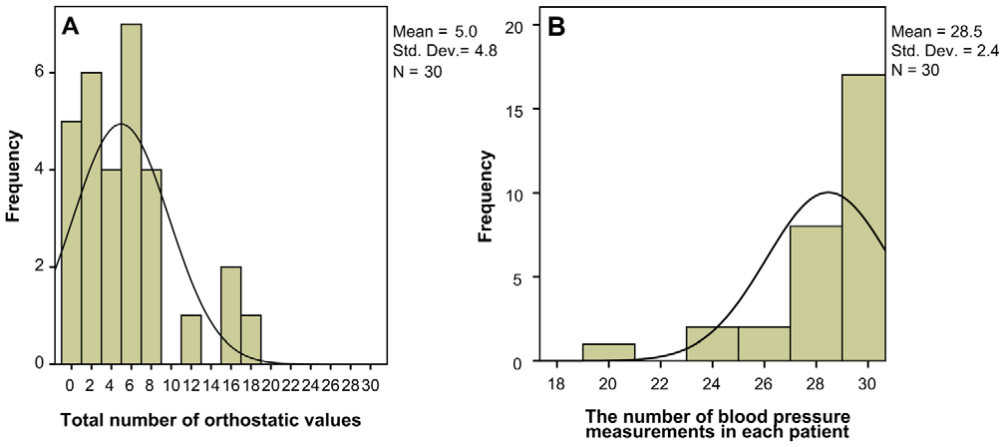
a. Orthostatic values. Variance in the total number of orthostatic values among the 30 patients. The median number was 4, 5 values out of maximum 30 values. **b. Blood pressure measurements.** The variance in the number of blood pressure measurements (median 30 (20–30) performed by each patient. All 30 patients were tested on at least three occasions, but not everybody was able to remain in standing position for 10 minutes. Therefore we could not always collect the maximum number of measurements (five systolic and five diastolic values).

To illustrate the extent of orthostatic hypotension, each patient's systolic and diastolic blood pressure values were analyzed individually. Each measurement point was dichotomized as orthostatic or not orthostatic. The sum of all orthostatic values (5 measure points at 3 assessments, systolic and diastolic, i.e. max 30 values) in each patient was calculated. Figure 2a illustrates the variance in the number of orthostatic values. The median number of orthostatic values was 4, 5 (range 0–18). To dichotomize the population into two groups we therefore used the cut off 4, which also was the recommended cut off according to a ROC curve analysis. Five or more orthostatic values were defined as persistent orthostatic hypotension.

Not everybody was able to remain in standing position for 10 minutes. Figure 2b shows the variance in the number of blood pressure measurements (median 30 (20–30)) performed by each patient.

### Urinary incontinence and constipation

At baseline all patients completed a thorough clinical examination including their medical history and concomitant medication. Cardiovascular medication was defined as drugs included in ATC group C, i.e. antihypertensives, diuretics, beta blockers, calcium channel blockers, renin-angiotensin II receptor blockers and treatment of hyperlipidemia. Information about continence was acquired through the disability assessment for dementia (DAD) [17]. Two questions are posed to the patient and caregiver considering 1) the ability to decide to use the toilet at appropriate times and 2) the ability to use the toilet without “accidents”. The presence of urinary incontinence was defined as a negative answer to any of these two questions. The presence of constipation was defined out of regular use of purgatives and/or enemas.

### Statistics

Statistical analyses were performed using PASW Statistics 18. All binary variables were compared using Chi-Square Tests. Due to the small number of subjects in this study, non-parametric statistics (Mann-Whitney U-test) were used to detect significant differences in groups for normally and non-normally distributed continuous variables. Since orthostatic hypotension was the most common autonomic disturbance and was assessed with the most systematic measurement, we explored the effect of persistent orthostatic hypotension on survival separately. To assess a possible dose-effect, we also studied the effect of the additional presence of urinary incontinence and/or constipation. Kaplan–Meyer curves were performed to compare survival in two groups. The p values were two sided exclusively and level of significance defined as less than 0.05.

### Ethics statement

The RCT was conducted in accordance with the Declaration of Helsinki as revised in 2000 and approved by the regional ethics committee at Lund University, as was the open-label extension study. Renewed written informed consent was obtained from patients and their caregivers before entering the open-label extension.

## Results

**Figure 3:**
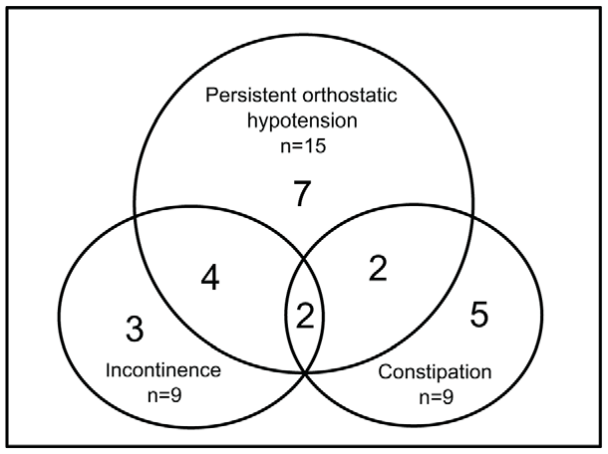
Dysautonomic features in our patient sample. Frequency of the three most common features of autonomic dysfunction, persistent orthostatic hypotension, urinary incontinence and constipation. The total number of patients was 30 and the most common manifestation was orthostatic hypotension. 15 patients (50%) had persistent orthostatic hypotension. Incontinence and constipation were equally common; 9 patients (30%) in each group. 7 patients (23%) had persistent orthostatic hypotension, but no other manifestation. 8 patients (27%) had persistent orthostatic hypotension and one or more manifestation. 2 patients had all three manifestations. 7 patients (23%) had no manifestations of autonomic dysfunction.

The frequency of symptoms related to autonomic dysfunction in this population is described in figure 3. 25 (83%) of the patients had by definition orthostatic hypotension (i.e. at least one measurement with orthostatic hypotension) and 15 (50%) had persistent orthostatic hypotension (five or more orthostatic values). Urinary incontinence and constipation were equally common (30%).

Comparisons were made between the groups with and without persistent orthostatic hypotension (Table 1). The median age at baseline was equal in both groups. Gender distribution was not significantly different, but the male preponderance tended to be more pronounced among the patients with persistent orthostatic hypotension. MMSE score and disease duration were equal. Cancer, heart disease, coexisting vascular risk factors and the use of cardiovascular medication were equally common. The use of Cholinesterase inhibitors and antidepressants were equal in both groups. SSRI was the most common type of antidepressant (50%). The median daily dose of Levodopa was equal.

**Table 1:** Demographics.

	Persistent orthostatic hypotension	No or mild orthostatic hypotension	p
Total number of subjects	15	15	
Age (median (range))	75 (63–82)	75 (68–82)	0.90
Gender (m/f (%f))	14/1 (6%)	9/6 (40%)	0.08
Diagnosis (DLB/PDD)	8/7	8/7	1.00
Treatment (placebo/memantin)	8/7	6/9	0.72
MMSE score (median (range))	20(14–26)	23 (13–26)	0.39
Disease duration	7(1–12)	5 (1–11)	0.53
Diabetes (% yes)	2/15 (13%)	1/15 (7%)	1.00
Hypertension (%yes)	2/15 (13%)	4/15 (27%)	0.65
Heart disease (%yes)	5/15 (33%)	6/15 (40%)	1.00
Cancer (%yes)	1/15 (7%)	1/15 (7%)	1.00
Heart and antihypertensive medication (%yes)	10/15 (67%)	9/15 (60%)	1.00
Antidepressants (% yes)	8/15 (53%)	5/15 (33%)	0.46
Cholinesterase inhibitors (% yes)	10/15 (67%)	6/15	0.27
Levodopa dose (mg/day)	450 (25–850)	550 (25–1700)	0.80

Demographics and clinical features at baseline. Comparisons are made between patients with and without persistent orthostatic hypotension.

### Survival

Seven (23%) of the 30 patients died during the follow up, 5 from the DLB group and 2 from the PDD group.

**Figure 4:**
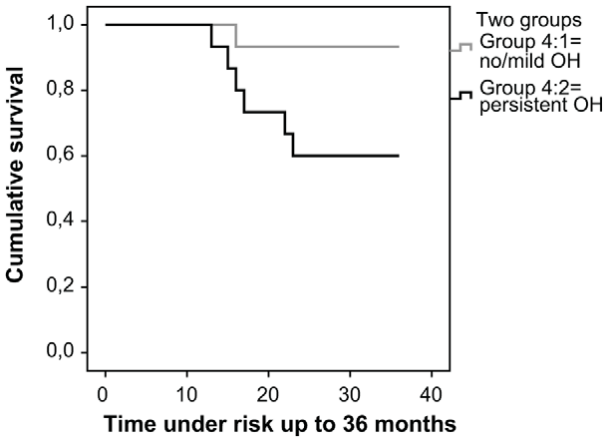
Orthostatic hypotension and survival. In this Kaplan-Meyer curve we wanted to test if persistent orthostatic hypotension affected survival. Concurrent manifestations of autonomic dysfunction (constipation and urinary incontinence) were not taken into consideration. The black line (group 4∶1) represents the 15 patients with persistant orthostatic hypotension and the grey line(group 4∶2) represents the 15 patients with no or mild orthostatic hypotension (se figure 3). No censored cases. Survival analysis showed that patients with persistent orthostatic hypotension had a significantly shorter survival compared to these with no or mild orthostatic hypotension. Log rank x^2^ = 6.370, p = 0.041.

The Kaplan Meyer curve in figure 4 shows the influence of orthostatic hypotension on survival. Patients with persistent orthostatic hypotension had a significantly shorter survival (Log rank x^2^ = 4.47, p = 0.034). There were no significant differences in survival between patients with and without constipation or with and without urinary incontinence. (Data not shown).

**Figure 5:**
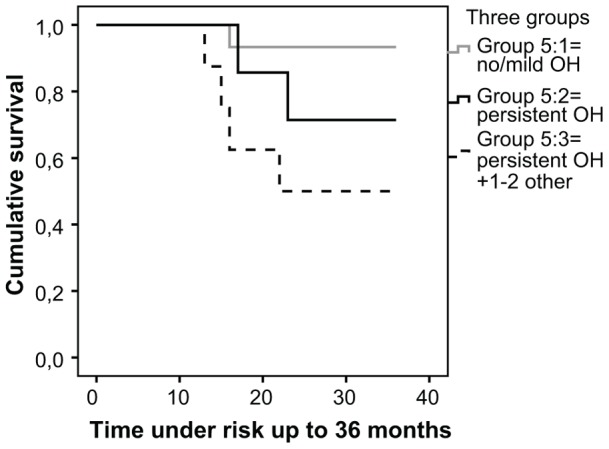
Manifestations of autonomous dysfunction and survival. In this Kaplan-Meyer curve we divided the patients in three groups, aiming to test if presence of concurrent manifestations of autonomous dysfunction, ie constipation and urinary incontinence in addition to persistent orthostatic hypotension, had an adding negative effect om survival. The grey line (group 5∶1) represent the 15 patients with no or mild orthostatic hypotension. The black line (group 5∶2) represent the 7 patients with severe orthostatic hypotension but no other manifestation. The black dashed line (group 5∶3) represent the 8 patients with severe orthostatic hypotension and one or two more manifestations. No censored cases. Survival analysis showed a significant difference between the three groups (Log rank x^2^ = 6.370, p = 0.041), where patients with one or two more manifestations in addition to persistent orthostatic hypotension (group 5∶3) had the shortest survival and group 5∶2 had the next shortest survival.

When we divided the patients in three groups (figure 5), where group 1 (n = 15) had no or mild orthostatic hypotension, group 2 (n = 7) had isolated persistent orthostatic hypotension and group 3 (n = 8) had persistent orthostatic hypotension together with constipation and/or urinary incontinence, we found a significant difference between the three groups (Log rank x^2^ = 6.370, p = 0.041). Patients in the third group had the shortest survival and the second group had the next shortest (Figure 5). The two patients with all three manifestations (persistent orthostatic hypotension, urinary incontinence and constipation) had the shortest survival (data not shown).

The 12 patients out of 42 in the Swedish population that did not complete the three assessments (Figure 1), were older (median age 79 and 75 respectively, p = 0.03). There were no differences in MMSE score at baseline or blood pressure in supine position. The fraction of deceased after 36 months was equal among the drop outs compared to the completers.

## Discussion

This is the first study to analyze the influence of autonomic dysfunction on survival in patients with DLB. We show that presence of persistent autonomic dysfunction is a possible predictor of a shorter survival in this population. The frequency of symptoms related to autonomic dysfunction was high in this DLB cohort and the most frequent manifestation was orthostatic hypotension.

In PD, non-motor symptoms like autonomic system dysfunction, depression, psychosis and sleep disturbances are considered by many patients to be more disabling than the motor symptoms [18], [19]. Despite this, they are often poorly recognized and inadequately treated, in contrast to the motor symptoms of the disease. [20], [21], [22]. Patients with DLB and PDD report a more impaired quality of life compared to AD [23]. Given that non-motor symptoms, including autonomic dysfunction, have a notable impact on quality of life in PD [20], this may be the case also in DLB.

There are different methods to evaluate the presence and severity of autonomic dysfunction. Many earlier studies on different diagnostic populations have a retrospective study design and they share the unreliable nature of such data. Patients may not report symptoms of autonomic dysfunction spontaneously, and the prevalence is most likely underestimated. Although orthostatic hypotension is known to give symptoms like light-headedness, visual blurring, dizziness, generalized weakness, fatigue, coat-hanger ache, nausea and abdominal discomfort it is shown that only 43% of non-demented patients with profound orthostatic hypotension have typical symptoms [24]. Corresponding findings are reported for demented patients [25]. The great majority of recent studies on PD use scales based on reports from patients and caregivers to detect non-motor symptoms. We have found only one scale where presence of orthostatic hypertension is based on objective blood pressure measurements; Composite Autonomic Severity Score (CASS) [26], [27].

In this prospective study we focus on the three most common [28], [29] dysautonomic symptoms; constipation, urinary incontinence and orthostatic hypotension. A strength of the study is that orthostatic hypotension was detected by repeated and standardized blood pressure measurements. We do not specify whether patients with orthostatic hypotension are asymptomatic or symptomatic. In most studies, the time of standing during an orthostatic test is 3–5 minutes. Importantly, demented patients may not show significant falls in blood pressure until as late as after 10 minutes [25], [30]. Measurements of blood pressure should therefore be recorded for at least 10 minutes after standing up. It has also been shown that DLB patients react different to orthostatic challenge compared to AD and controls, with a more prolonged period of orthostasis after standing up [31]. All patients in our study had three documented orthostatic tests during the follow up, although a few did not manage to accomplish the 10 min measure point. The 12 patients who did not complete the three assessments were excluded, which means that there is a possibility that those with the most severe orthostatic hypotension are not in the study. This may strengthen our results.

Our results suggest that orthostatic hypotension is the more important prognostic factor compared to constipation and incontinence as neither of these two manifestations alone was found to affect survival. We have not found any earlier studies addressing this issue. A possible explanation is that constipation and urinary incontinence are common in an elderly population due to a variety of causes and their specificity for autonomic dysfunction is probably low, especially when they exist without concurrent orthostatic hypotension. However, there are also several possible explanations to orthostatic hypotension. Common reasons in the elderly are dehydration and congestive heart failure, but also side effects from drugs, for example antihypertensive medication, antiparkinson medication or certain antidepressants. The negative effect of orthostatic hypotension on survival may be independent of the underlying cause of orthostatic hypotension. In this study, information on constipation and urinary incontinence is based on prospectively collected reports from patients and caregivers but no objective test. Furthermore, we did not rate the severity, as we did with orthostatic hypotension. The major limitation of our study is the small sample size. Due to this, and especially the low number of events (n = 7) a multivariate analysis to adjust for possible confounders, was not feasible. In our population with DLB/PDD patients, the frequency of coexisting heart disease and the use of antihypertensive and antiparkinson medication were equal in both groups. There was no significant difference in the use of antidepressants between groups, but it tended to be more common in the subgroup with persistent orthostatic hypotension. It is possible that the more severe orthostatic hypotension is at least partly influenced by concomitant medication.

The pathophysiological mechanism of the association between autonomous dysfunction and mortality is not clear. Autonomic dysfunction is thought to be related to the α-synuclein pathology, since several neuropathological studies describe the presence of lewy bodies in strategic locations like locus coeruleus, sympathetic ganglia and parasympathetic plexus [32]. In PD, the non-motor symptoms are related to advanced stages in patients with a fully developed motor phenotype [33], but are reported significantly more common in the disease across all stages than in controls [34]. The latency from onset of disease to orthostatic hypotension seems to be delayed in DLB compared to MSA [28], [35] and PD [28].

Studies comparing survival in AD and DLB are inconclusive. Some studies report a similar rate of cognitive decline, but a shorter survival [36], [37] and shorter time to institutionalization in DLB [7], [38]. These findings suggest that progression of non-cognitive symptoms differ between AD and DLB, and our findings add to these findings by suggesting that autonomic dysfunction may be one of the contributing factors to this difference.

The extent and pattern of autonomic dysfunction in dementia with Lewy bodies are poorly documented, even though many authors mention it as a well-known clinical feature. To our knowledge, this is the first study to investigate its influence on prognosis and survival in DLB even though there are a few studies proposing that repeated hypotensive episodes may exaggerate cognitive decline on the basis of cerebral hypoperfusion and microvascular lesions [39], [40]. However, further research is needed to clarify the impact of autonomic dysfunction on factors such as than cognition, quality of life, activities of daily living and behavioral and psychiatric symptoms.

In summary we have found a high frequency of symptoms related to autonomic dysfunction in patients with DLB/PDD and importantly, patients with persistent orthostatic hypotension had a shorter survival. This needs to be verified in larger studies. Orthostatic hypotension seems to be the most important feature in autonomic dysfunction and we strongly recommend orthostatic blood pressure measurement on a routine basis in all patients with DLB/PDD.
